# Talcum induced pneumoconiosis following inhalation of adulterated marijuana, a case report

**DOI:** 10.1186/1746-1596-7-26

**Published:** 2012-03-15

**Authors:** Andreas Hans Scheel, Daniel Krause, Helmut Haars, Inge Schmitz, Klaus Junker

**Affiliations:** 1 Institute of Pathology, Klinikum Bremen-Mitte, St.-Jürgen-Str. 1, 28177 Bremen, Germany; 2 Department of Pneumology and Respiratory Medicine, Klinikum Bremen-Ost, Züricher Str. 40, 28325 Bremen, Germany; 3 Institute of Pathology, Ruhr-Universität Bochum, Berufsgenossenschaftlichen Universitätsklinik Bergmannsheil, Bürkle-de-la-Camp-Platz 1, 44789 Bochum, Germany; 4 Department of Molecular Oncology, University Medicine Göttingen, Justus-von-Liebig-Weg 4, 37077 Göttingen, Germany

**Keywords:** Pneumoconiosis, Talcosis, Interstitial lung disease, Drug abuse, Cannabis, Energy dispersive x-ray spectroscopy

## Abstract

**Background:**

Talcosis, a granulomatous inflammation of the lungs caused by inhalation of talcum dust, is a rare form of pneumoconiosis. Besides inhalative occupational exposure, intravenous abuse of adulterated drugs is a major cause for this condition. Minerals such as talcum (magnesium silicate) and sand (predominant silicon dioxide) are used to increase both volume and weight of illicit substances. In intravenous heroin-abuse, talcosis is a well-known complication. Here we describe a case of talcosis caused by inhalative abuse of adulterated marijuana.

**Clinical history:**

A 29-year old man presented with persistent fever, dyspnea and cervical emphysema. He admitted consumption of 'cut' marijuana for several years, preferentially by water pipe smoking.

**Morphologic findings:**

Lung-biopsies showed chronic interstitial lung disease, anthracotic pigments and birefringent material. Energy dispersive x-ray spectroscopy revealed silicon-containing particles (1-2 μm) and fine aluminum particles (< 1 μm), magnesium and several other elements forming a spectrum compatible with the stated water pipe smoking of talcum-adulterated marijuana.

**Conclusions:**

The exacerbated chronic interstitial lung disease in a 29-year old patient could be attributed to his prolonged abuse of talcum-adulterated marjuana by histopathology and x-ray spectroscopy. Since cannabis consumption is widely spread among young adults, it seems to be justified to raise attention to this form of interstitial pulmonary disease.

**Virtual slides:**

The virtual slide(s) for this article can be found here: http://www.diagnomx.eu/vs/krause/html/start.html.

## Background

Pneumoconioses are restrictive pulmonary diseases caused by chronic inhalation of mineral dust. In many industrialised countries, improvements in occupational health lead to a general decrease of pneumoconiosis over the last 5 decades. Contrary to this trend, the incidence of asbestosis strongly increased until a few years ago rendering it the currently most frequently recorded pneumoconiosis [1]. While talc (hydrated magnesium silicate) is commonly used in various industrial processes, its clinical relevance (Talcosis, ICD-10 J23.0) is fairly low. In addition to chronic inhalation, intravenous drug abuse is a major source of talc-related lung diseases: As talc is used as filling material in tablets, intravenous abuse of drugs intended for oral application causes deposition of the mineral in various organs, notably the lungs [2]. Illicit substances such as heroin are often adulterated to increase volume and weight for which talc is frequently used [3].

Talc deposition in the lungs typically causes granulomatous inflammation characterized by formation of foreign body granulomas of varying degree within a fibrotic stroma. These granulomas are typically composed of free or intracellular birefringent deposits accompanied by multinucleated giant cells. They may also appear ill defined with only few surrounding histiocytes. Distribution of these lesions is variable and they may develop intra- and perivascular as well as in the interstitium.

Radiologic findings include micronodular patterns of diffuse or well defined nodules which may fuse with progression to form larger opacities in the perihilar regions. Other manifestations include interstitial thickening and lower-lobe emphysema. High-resolution computed tomography may show small centrilobular nodules or ground-glass opacities presumably representing microscopic granulomas below tomography resolution. With progression of the disease heterogeneous masses with internal foci of high attenuation resembling talc deposition may develop [4].

Reports on adulteration of cannabis are rare and just one publication describes the consequences of inhalative abuse of cannabis adultered with mineralic substances [5]. Here we present the case of a 29-year-old patient who developed chronic interstitial pulmonary disease most likely due to long-term smoking of marijuana adulterated with mineral substances. The histological diagnosis was confirmed by energy dispersive x-ray spectroscopy which yielded a spectrum of elements compatible with talcum as the leading contaminant.

## Case presentation

### Initial clinical presentation

The 29-year old patient was admitted to hospital treatment with persistent fever (40°C), dyspnea with wet cough and cervical emphysema. The symptoms had been present for six days. Oral antibiosis (ciprofloxacin 2 × 500 mg/d) had been prescribed prior to admission. The histological findings following transbronchial biopsies led to the initial conclusion of the interstitial pulmonary disease being caused by intravenous abuse either of adulterated illicit substances or of dissolved tablets. Taken the fact into account of locally available cannabis being commonly adulterated with substances such as sand and talcum we reevaluated the lung biopsies and performed energy dispersive x-ray spectroscopy (EDX).

### History and clinical examination

Examination of the patient revealed no further pathologic findings. In particular, auscultation of the lung and heart was within normal limits and percussion indicated regular lung margins. The patient admitted regular consumption of cannabis by water-pipe smoking for several years but denied abuse of any other drugs. There were no signs of continued intravenous drug abuse nor evidence of any occupational exposure.

### Imaging

**Figure 1:**
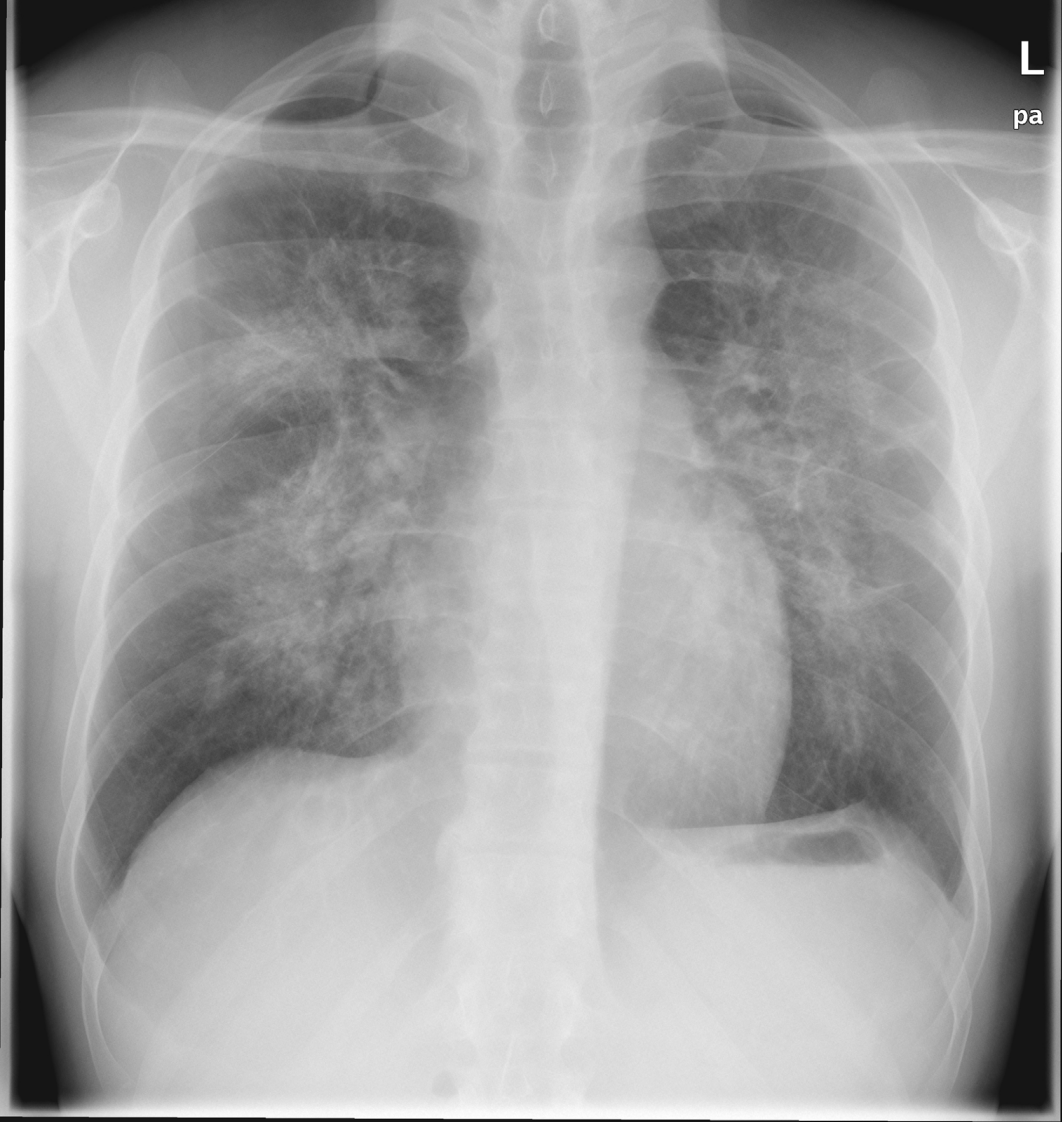
**Chest x-ray showing centrally pronounced bilateral pulmonary infiltrates with ground-glass opacities**.

**Chest x-rays **revealed ubiquitous, centrally pronounced infiltrates [Figure 1]. **High-resolution computed tomography scans **confirmed the cervical emphysema which extended into the mediastinum. Given the absence of bullae and other specific radiologic findings, the emphysema was considered as caused by the persistent coughing. Furthermore, the scans showed bilateral peribronchovascular alveolar shadows with groundglass-appearance.

### Clinical investigations

Upon admission **blood tests **showed high c-reactive protein (145 mg/L) and moderate leukocytosis (15000/μL). **Arterial blood gas **analysis indicated partial respiratory insufficiency (pO2 57.3 mmHg, pCO2 35.5 mmHg, pH 7.465). **Spirometric studies **were suggestive of restrictive lung disease (VC 3.73 L = 73%, FEV1 2.94 L = 71%, FEV1/VCmax 79, RAWtot 46%, TLC 4.98 L = 74%, RV 1.25 L = 76%). **Bronchoalveolar lavage **revealed slightly increased absolute cell numbers of 16/μL with relative increase of neutrophils (13%) as well as macrophages containing large amounts of anthracotic pigments compatible with heavy smoking. Aerobic and anaerobic cultures yielded no pathologic growth. Negative polymerase chain reaction for mycobacterium tuberculosis was subsequently confirmed by negative cultures. **Immunological screening **for various specific IgG-antibodies was negative (i. e. pigeon, budgerigar, aspergillus fumigates and versicolor) rendering an extrinsic allergic alveolitis unlikely. A urine quick-test for legionella antigens was negative. The patient was tested negative for HIV and hepatitis C. Immunofluorescence microscopy for pneumocycstis jiroveci was negative.

### Treatment

The antibiotic treatment was switched to a combination of clarithromycin 2 × 500 mg/d per os and ceftriaxon 2 g/d intravenously. Under this regime the clinical condition of the patient improved while the leukocytosis and c-reactive protein were declining. However, the chest x-rays showed persistent infiltrates. A second bronchoscopy was performed and biopsies were taken.

Upon finding of birefringent material the therapy was supplemented by prednisone 30 mg/d per os. The clinical condition of the patient further improved and he could be released in good health on day 14. A final chest x-ray indicated a slight decrease of the pulmonary infiltration, stepwise reduction of the prednisone and follow-up chest x-rays were recommended.

### Histopathology and energy dispersive x-ray spectroscopy

**Figure 2:**
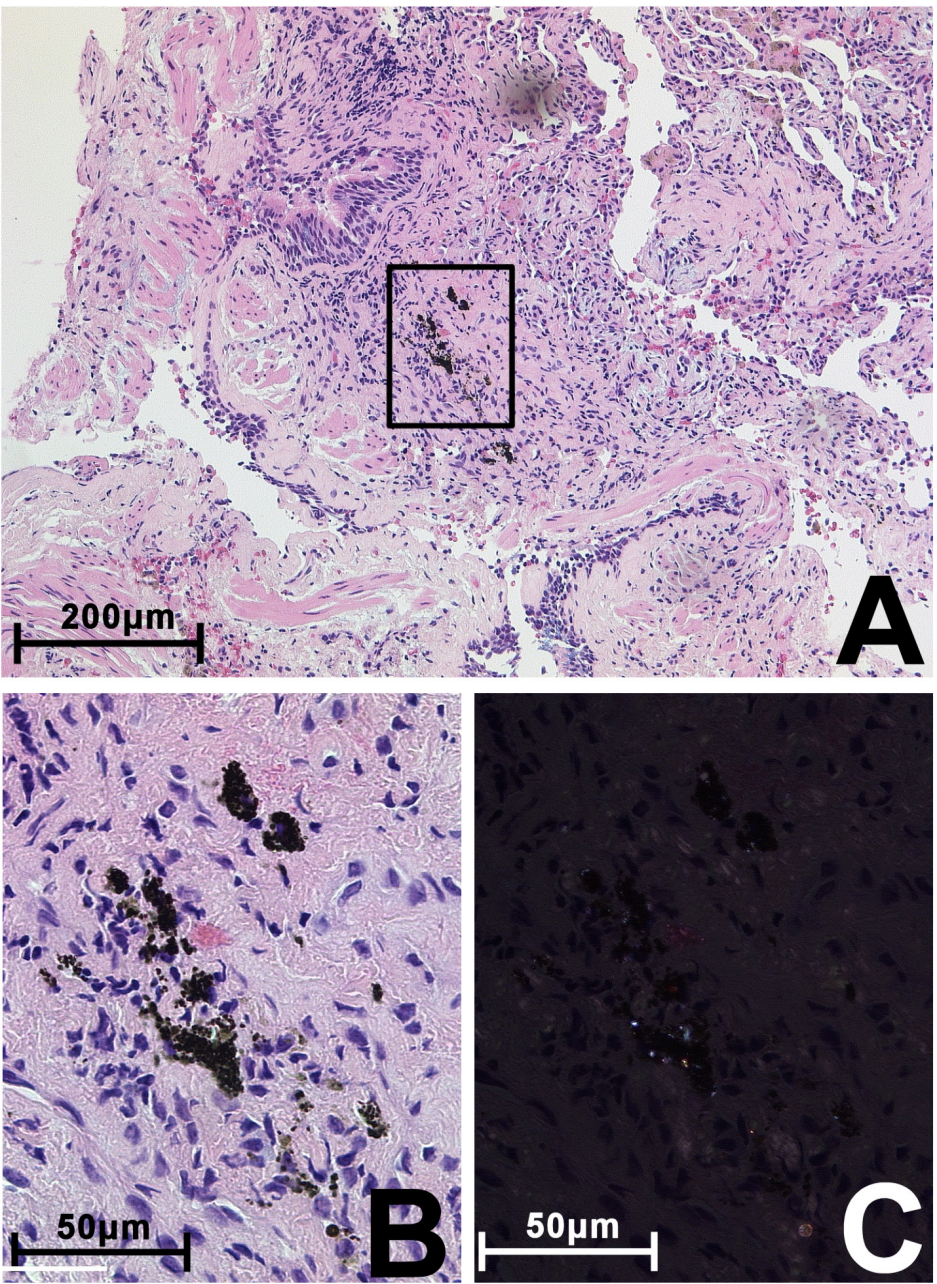
**Lung biopsy showing interstitial accented chronic inflammation and anthracotic pigment containing fine birefringent material**. **A**: Overview, HE staining. Scale bar = 200 μm, enlarged area highlighted (black box). **B**: Enlargement of A showing the anthracotic pigment and surrounding reactive tissue, scale bar = 50 μm. **C: **Same area as B illuminated by polarised light: Bright deposits of birefringent materials and dimly glowing collagen fibers.

Histopathological examination of transbronchial biopsies from the right upper and lower lobe using hematoxylin and eosin staining revealed chronic as well as active bronchitis together with active peripheral bronchiolitis and murally emphasised alveolitis. These findings were consistent with interstitially accented chronic pneumonia with transition in chronic organising pneumonia with patchy scarring. Alveolar hemorrhage and dust laden macrophages were present within the alveolar spaces and in the interstitium loosely surrounded by lymphocytes. Anthracotic pigment and fine birefringent crystal deposits ranging from 1 μm to 2 μm in size were detected by polarized light [Figure 2]. Similar birefringent particles were found inside alveolar macrophages. No foreign material was seen in the capillaries of the alveolar walls. No intravascular granulomas or obliterative thrombi were present. Methenamine silver and acid fast staining (Ziehl-Neelsen) did not reveal any mycobacteria or fungi.

**Figure 3:**
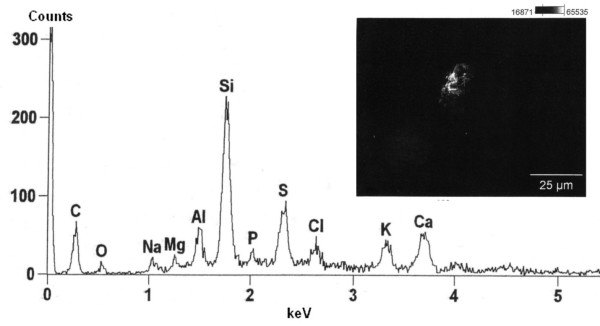
**Energy dispersive x-ray spectroscopy showing Silicon as major element (Xaxis: Energy of detected x-ray quanta in keV; Y-axis: Signal-intensity in counted x-ray quanta; Elements corresponding to the intensity-peaks indicated by there element symbols)**.

The birefringent crystals were analysed by energy dispersive x-ray spectroscopy (EDX). Silicon was identified as the major element of the 1 μm to 2 μm particles along with sulfur, carbon and calcium [Figure 3]. Magnesium, sodium and traces of copper were also detected. Besides the crystaline deposits, tiny (< 1 μm) aluminum particles were also found.

## Discussion

The EDX spectrum showing silicium and magnesium confirms the presence of materials being used for adulteration, in particular talc. Other elements detected by EDX spectroscopy are in keeping with the suspected inhalative exposure. Magnesium may be as well related to magnesium enriched charcoal which enhances inflammability and thus makes handling more convenient. The aluminum particles might be attributed to the stated long term water pipe smoking since common design of pipes features a layer of aluminum foil covering incinerated charcoal and providing a surface for a stack of tobacco. Using enhanced charcoal and aluminum foil containing water pipes as described are both common practice.

A typical EDX-spectrum of talcosis can be found in a recent case report of occupational talcosis by Neumann et al. [6]. Silicon, magnesium and oxygen are identified as leading elements with almost no impurities by other elements.

Although the clinical and histological findings presented in this case cannot be regarded as specific, elemental analysis of the birefringent particles in context with the patient's history can establishes the diagnosis of talcosis. In differentiating inhalation talcosis from pulmonary disease due to intravenous administration the distribution of inflammatory changes may be helpful with vascular related lesions tending to develop more commonly in talcosis induced by intravenous application. Furthermore, to distinguish inhalation talcosis from intravenous injection the size of the particles has been postulated to be a discriminator. Particles with a mean diameter of less than 5 μm are found most commonly in cases of inhalation talcosis while intravenous exposure has been reported to cause deposition of larger particles [7]. The fine deposits found in the biopsies investigated in this case make intravenous abuse unlikely and point towards inhalative exposure.

In comparison to interstitial lung disease due to intravenous administration of talc during drug abuse talc inhalation is a rare form of pneumoconiosis most often caused by occupational exposure. This may be attributed to its low fibrogenic potential rather than to infrequent exposure as talcum is widely used in various industrial processes and products. It has been described to induce fibrosis to a much lesser extent compared to other minerals such as quartz and asbestos [8]. Still, talcum has the potency to initiate fibrosis on its own and extent of exposure and latency of fibrosis is dose-dependent. The biopersistency of talcum seems to be high with deposits identifiable decades after exposure [6]. The non-occupational inhalative exposure to talc due to long-term smoking of adulterated cannabis and subsequent development of pulmonary disease as described has been rarely recognized so far. While talcosis is a recognized complication in abuse of heroin and intravenous consumption of drugs prepared for oral application, adulterated cannabis is far less investigated. Only one case report from France covering the consequences of adulterated cannabis has been published so far [5]. Delourme et al. describe two patients who consumed cannabis adulterated either by glass beads or with sand; interestingly computed tomography of the lungs revealed ground glass opacities in the later, just like in this case. The French Department of Health subsequently issued an advice to consumers regarding the risks of adulterated cannabis. Cannabis consumption is a major health issue in the European Union. The lifetime prevalence for occasional cannabis-use among 18-22 year old adolencents in Germany was estimated to be 40% in 2010. In the same survey, 3% of the 7000 participants admitted regular cannabis consumption. This corresponds to approximately 250 000 individuals if projected to the German population in that age group [9]. Lung diseases caused by adulteration substances may thus be more common than is reflected by current literature, especially since drug-abuse may not admitted by a patient.

## Conclusions

The exacerbated chronic interstitial lung disease in a 29-year old patient could be attributed to his prolonged abuse of adulterated marjuana by histopathology and x-ray spectroscopy. Both size and elemental composition of mineralic deposits found in biopsies were compatible with inhaled talcum as the leading contaminant.

Given the high numbers of cannabis users it seems to be justified to raise attention to this form of interstitial pulmonary disease. It may be included as a differential diagnosis in evaluating chronic pulmonary disease in young adults. If cannabis consumption is suspected, the patient should be informed about the additional risks of adulterated cannabis, particularly owing to the fact of talcum having a long biopersistency.

## Competing interests

The authors disclose financial competing interests.

The authors declare that they have no competing interests.

## Authors' contributions

HH was the responsible clinician for the reported patient. KJ, DK and AHS performed histopathologic examination of the biopsies. X-Ray spectroscopy and evaluation of the obtained data was performed by IS. The manuscript was drafted by AHS, DK and KJ. AHS and DK contributed equally to this work and would like to share first-authorship. All authors read and approved the final manuscript.

## Authors' information

KJ is certified professor of pathology and head of the of the institute of pathology at the hospital 'Bremen-Mitte', Bremen, Germany. He was promoted professor of pathology at University of Bochum in 2004 and is specialised in malignancies of the lungs. The University hospital of Bochum and it's institute of pathology have a focus on lung diseases given that the circumjacent 'Ruhrgebiet'-area has been a center of mining and steel industry for decades. The institute directs the German mesothelioma register.

AHS and DK are medical doctors and pathologists in training who worked at KJ's institute while the manuscript was written.

HH is medical doctor at the department of pneumology and respiratory medicine, hospital 'Bremen-Ost', Bremen, Germany. He is specialised in obstructive and infectious lung diseases.

IS is scientist (Dr. rer. nat.) at the institute of pathology at the University hospital in Bochum and responsible for physical/chemical characterisation of materials. The institute offers a broad spectrum of specialised techniques including electron microscopy, x-ray spectroscopy and ashing for quantification of asbestos fibres. IS has a record of scientific publications in this field.
